# CFTR Protein: Not Just a Chloride Channel?

**DOI:** 10.3390/cells10112844

**Published:** 2021-10-22

**Authors:** Laurence S. Hanssens, Jean Duchateau, Georges J. Casimir

**Affiliations:** 1 Department of Pediatric Pulmonology and Cystic Fibrosis Clinic, Hôpital Universitaire des Enfants Reine Fabiola, Université Libre de Bruxelles (ULB), Avenue J.J. Crocq 15, 1020 Brussels, Belgium; georges.casimir@huderf.be; 2 Laboratoire Académique de Pédiatrie, Hôpital Universitaire des Enfants Reine Fabiola, Université Libre de Bruxelles (ULB), Avenue J.J. Crocq 15, 1020 Brussels, Belgium; jeanduch@gmail.com

**Keywords:** cystic fibrosis, CFTR protein, channel, chloride, bicarbonate, glutathione, thiocyanate, macrophages, neutrophils, lipids

## Abstract

Cystic fibrosis (CF) is a recessive genetic disease caused by mutations in a gene encoding a protein called Cystic Fibrosis Transmembrane Conductance Regulator (CFTR). The CFTR protein is known to acts as a chloride (Cl^−^) channel expressed in the exocrine glands of several body systems where it also regulates other ion channels, including the epithelial sodium (Na^+^) channel (ENaC) that plays a key role in salt absorption. This function is crucial to the osmotic balance of the mucus and its viscosity. However, the pathophysiology of CF is more challenging than a mere dysregulation of epithelial ion transport, mainly resulting in impaired mucociliary clearance (MCC) with consecutive bronchiectasis and in exocrine pancreatic insufficiency. This review shows that the CFTR protein is not just a chloride channel. For a long time, research in CF has focused on abnormal Cl^−^ and Na^+^ transport. Yet, the CFTR protein also regulates numerous other pathways, such as the transport of HCO_3_^−^, glutathione and thiocyanate, immune cells, and the metabolism of lipids. It influences the pH homeostasis of airway surface liquid and thus the MCC as well as innate immunity leading to chronic infection and inflammation, all of which are considered as key pathophysiological characteristics of CF.

## 1. Introduction

Cystic fibrosis (CF) is the most common life-shortening genetic disease in Caucasian people [1,2,3]. Since its first description in the 1930s [4,5], its pathophysiology has become better understood. CF is a recessive disease resulting from mutations in a single gene that encodes a protein called Cystic Fibrosis Transmembrane Conductance Regulator (CFTR). Following the discovery of this gene in 1989, the CFTR protein structure and function have been largely described.

The CFTR gene is expressed on chromosome 7 (7q31.2). More than 2000 gene variants have been identified so far. These mutations have different effects on the CFTR protein synthesis, function, and stability at the cell membrane [1]. They have been classified into six or even seven classes [1,6], which are essential for understanding the pathophysiology and developing new therapeutic approaches for CF, such as the CFTR modulators that increase qualitatively and quantitatively the CFTR protein activity.

**Figure 1:**
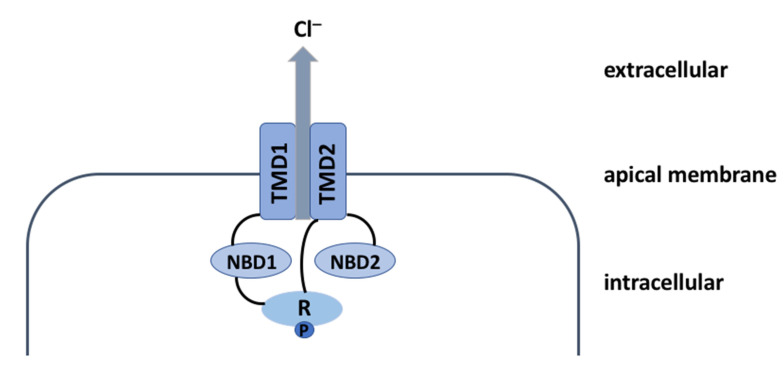
Schematic representation of CFTR protein. CFTR protein includes several domains: two transmembrane domains (TMD1 and TMD2), two cytosolic nucleotide-binding domains (NBD1 and NBD2), and a single regulatory R-domain. TMD1 is linked to NBD1 and TMD2 is linked to NBD2, thereby forming two TMD-NBD complexes united by the R-domain. The TMDs form the channel of the CFTR protein, while the NBDs regulate its opening and closure. As the CFTR channel is an ATP-dependent ion channel, its opening requires R-domain phosphorylation (P) by the protein kinase A (PKA) and ATP binding at the NBDs leading to their dimerization, which in turn allows the chloride (Cl^−^) ions to exit the epithelial cells. Channel closure is triggered by ATP hydrolysis, which results in the separation of the NBD dimer and restoration of the TMD conformation [3]. Abbreviations: Cl^−^ = chloride; TMD = transmembrane domain; NBD = nucleotide-binding domain; R = regulatory domain; P = phosphorylation site.

The CFTR protein is a 1480-amino acid glycoprotein belonging to the adenosine triphosphate (ATP)-binding cassette (ABC) transporter superfamily. Its architecture is complex and it allows the chloride (Cl^−^) ions to exit the epithelial cells (Figure 1).

This Cl^−^ channel function of the CFTR protein in the epithelial cell apical membrane of the lungs, upper respiratory tract, pancreas, liver, gallbladder, intestines, sweat glands, and reproductive tract is essential for the osmotic balance of the mucus and its viscosity. Owing to this widespread distribution of the CFTR protein, CF is a multisystem disease (Table 1), although lung involvement, characterized by bronchial obstruction, infection, and inflammation leading to bronchiectasis and progressive pulmonary injury, remains the main cause of morbidity and mortality [7,8]. The CFTR protein is thus commonly known as a Cl^−^ channel expressed in the exocrine glands of several body systems where it also regulates other ion channels, particularly the epithelial sodium (Na^+^) channel (ENaC) that plays the central role in salt absorption. It also regulates the transport of bicarbonate (HCO_3_^−^), which was found to be largely involved in the pathophysiology of CF [9]. HCO_3_^−^ plays a major role in airway surface liquid (ASL) pH homeostasis and therefore in the innate immunity system and mucus viscosity. The CFTR protein is also expressed by immune cells, the loss of functional CFTR in CF may result in dysregulation of their functions. In addition, the CFTR protein influences the metabolism of lipids that are the main component of cell membranes. In CF, eicosanoid synthesis through the arachidonic acid (AA) pathway plays a crucial role in inflammation. Therefore, the pathophysiology of CF is more challenging than a mere dysregulation of epithelial Cl^−^ and Na^+^ transport in several body systems, mainly resulting in compromised mucociliary clearance (MCC) leading to pulmonary and pancreatic impairment. Indeed, these single gene mutations that compromise the CFTR function cause numerous metabolic changes, which explain the complexity of CF.

Even in the era of CFTR modulators considered as “life-changing drugs” for the CF patients, it is of paramount importance to understand the other functions of the CFTR protein and their implication in the pathophysiology of this disease. In addition to describing the main known function of the CFTR protein as a Cl^−^ channel, this review focusses on the relationships between the CFTR protein and the transport of HCO_3_^−^, glutathione (GSH), and thiocyanate (SCN^−^), as well as the immune cells and the metabolism of lipids that influence MCC and innate immunity and lead to chronic infection and inflammation, all of which are considered as key characteristics in the pathophysiology of CF.

## 2. The CFTR Protein: A Chloride Channel

**Figure 2:**
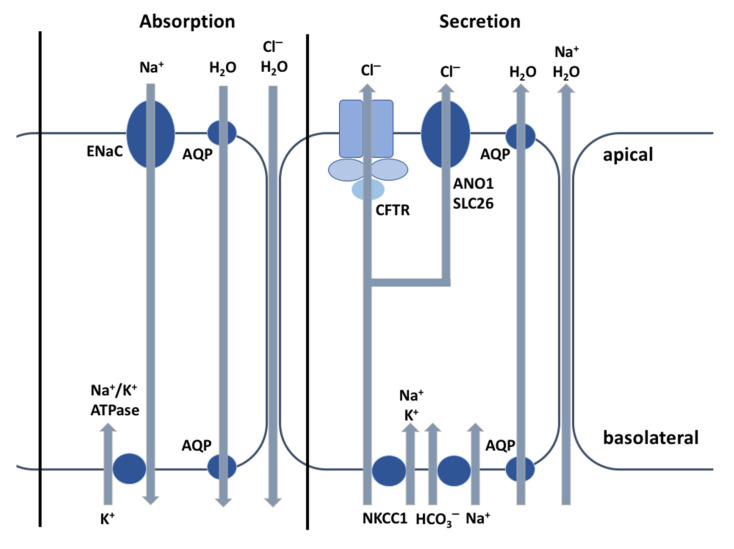
Schematic representation of ion transport in airway epithelia. Modified from Saint-Criq [12]. Epithelial tissues are made of one or more layers of cells that cover their surface and are joined to one another by junctional proteins. Epithelial cells are composed of two membranes with distinct functions depending on the tissue and on their ion channels, exchangers, cotransporters, or pumps. While the basolateral membrane is in contact with the interstitial tissue, the apical membrane is in contact with the external environment and thus also acts as a barrier to prevent potential pathogens or toxic compounds from reaching the bloodstream. Salt and water are absorbed or secreted via paracellular or transcellular pathways (respectively, ENaC and AQP). Cl^−^ secretion across the epithelial cell apical membrane usually occurs via CFTR, depending on the CFTR channel opening and number, and creates the driving force for Na^+^ secretion across the epithelial cells through the paracellular pathway, with water following osmotically via aquaporins or the paracellular pathway [12]. Abbreviations: CFTR = cystic fibrosis transmembrane conductance regulator; ENaC = epithelial sodium channel; ANO1 = anoctamin-1; AQP = aquaporins; NKCC1 = Na-K-Cl cotransporter; Cl^−^ = chloride; HCO_3_^−^ = bicarbonate; H_2_O = water; K^+^ = potassium.

Based on the observation that the sweat of CF patients is salty, which was first made by Paul di Sant’Agnese in 1953 [10], Gibson and Cooke developed in 1959 [11] the standardized sweat test by pilocarpine iontophoresis, which still remains the gold standard for diagnosing CF and demonstrating the associated Cl^−^ defect. The CFTR protein is mainly known as a Cl^−^ channel of the exocrine glands that interacts with other ions channels and is essential for the osmotic balance of the mucus and its viscosity. As shown in Figure 2, the CFTR plays a major role in electrolyte and fluid secretion and absorption. In CF, the Cl^−^ transport defect leads to many organ dysfunctions. In this review, we focus on CFTR protein function in the sweat glands and lungs, because of its crucial role in CF diagnosis and morbi-mortality.

In the sweat glands, the primary sweat, which is mainly secreted by the secretory coil of the eccrine glands, is isotonic with blood plasma. By creating an osmotic gradient, the salt in primary sweat is important for passive water secretion through the aquaporin-5 channel [13,14].

Due to the low water permeability of the epithelial cells lining the proximal part of the absorptive duct, only Na^+^ and Cl^−^ but not concurrent water are absorbed. This mechanism prevents salt loss from the body, which, if not controlled, could lead to circulatory collapse [12,15] and results in a hypotonic final sweat at the skin surface [14]. The transcellular reabsorption of Na^+^ and Cl^−^ from the lumen mainly depends on the amiloride-sensitive Epithelial Sodium Channel (ENaC) and CFTR in the apical membrane of epithelial cells, respectively. CFTR is expressed in both the secretory coil and absorptive duct of the sweat glands, though the highest levels are found in the duct cells. In the sweat glands, CFTR is involved in Cl^−^ secretion (primary sweat) and absorption (final sweat) [14]. However, non-CFTR channels, such as the calcium-activated chloride channel—also known as TMEM16A—or anoctamin-1 (ANO1), also contribute to Cl^−^ secretion, after being activated by an increase in cytosolic calcium (Ca^2+^) in response to cholinergic stimulation [16]. Na^+^ reabsorption results from the functional interaction between CFTR and ENaC [17], the ENaC channel being dependent on a functioning CFTR. In the basolateral membrane, intracellular Na^+^ is then pumped out of the cell into the interstitial fluid by the Na^+^/K^+^-ATPase pump (Figure 2). This generates a transepithelial electrical gradient favoring Cl^−^ absorption into the cell via CFTR and then Cl^−^ diffuses out of the cell through the basolateral CFTR channel. In contrast to all other CF-affected epithelial tissues, the CFTR protein in sweat glands is functionally present in both the apical and basolateral membranes, with a Cl^−^ flow reversal followed by Cl^−^ reabsorption. In CF, the loss of functional CFTR protein in both the basolateral and apical membranes of sweat ducts restricts Cl^−^ exit through the basolateral membrane and Cl^−^ reabsorption. This causes a significant depolarization of the apical membrane potential, thereby abolishing the electrical driving force and preventing passive diffusion of Na^+^. As a result, both Na^+^ and Cl^−^ ions are retained in the lumen and the final sweat excreted onto the skin surface contains higher Na^+^ and Cl^−^ concentrations (>60 mEq/L), this manifestation being considered as a diagnostic feature of CF [12]. Although this is not a major clinical problem per se, CF patients can lose excessive Na^+^, Cl^−^, and water in hot weather conditions (heat wave), which leads to severe hyponatremic dehydration if salt supplements are not taken.

Conducting airways are lined by a continuous epithelium and a thin (~10 μm) liquid layer (ASL). ASL mainly arises from submucosal gland secretions and transepithelial hydro-osmotic movements [18]. It comprises two distinct layers: the mucus layer (ML) and the periciliary layer (PCL). The ML contains large gel-forming mucins tethered to tips of the cilia, which trap inhaled particles or pathogens and propel them out of the lungs. The two main mucins in human airways are MUC5AC, primarily produced in the goblet cells of the surface epithelium, and MUC5B, mainly produced in the mucous cells of the submucosal glands [18]. The PCL is composed of 96% water, 1% salts, 1% lipids, 1% proteins, and 1% mucus. It is in direct contact with the epithelial cells and allows the cilia to beat [18,19]. CFTR is expressed at the apical membrane of airway ciliated epithelial cells and in submucosal glands. The recently identified ionocytes are a rare cell type (1–2% of epithelial cells), but they express the highest levels of CFTR protein (50% of CFTR transcripts) compared to the common ciliated epithelial cells (1.5% of CFTR transcripts) [20]. However, the precise role of ionocytes in Cl^−^ secretion remains unclear [18].

Effective MCC depends on the volume and composition of ASL, which is mainly controlled by two ion channels: CFTR and ENaC that regulate in concert the Cl^−^ and Na^+^ movements. In healthy airway epithelia, the CFTR protein normally secretes Cl^−^ at the epithelial cell apical membrane (Figure 2) and restricts Na^+^ reabsorption by ENaC downregulation. Short palate, lung, and nasal epithelial clone-1 (SPLUNC1) is an extracellular protein abundantly expressed in normal human respiratory epithelium. It binds to ENaC channel via an 18 amino acid domain on the protein’s *N*-terminus [21] and causes ENaC to internalize, thus preventing its activation by serine proteases [18]. SPLUNC1 inhibits ENaC channel and Na^+^ absorption [22] and, therefore, prevents ASL dehydration [23]. In CF, the loss of functional CFTR protein causes a defect in Cl^−^ secretion. SPLUNC1 is degraded by the neutrophil elastase that is present in high concentrations in the sputum and is ineffective at ASL pH values below 7.0 [19,24], leading to Na^+^ hyperabsorption [25]. Though CFTR is the main Cl^−^ channel in epithelial tissues, the non-CFTR Cl^−^ channels ANO1 and SLC26A9, present in the airway epithelium, also allow the apical secretion of Cl^−^ [26] and promote ASL hydration (Figure 2). A cross-activation between CFTR and these non-CFTR Cl^−^ channels has been demonstrated [27,28]. 

The high intracellular Cl^−^ and Na^+^ concentrations in CF lead to passive water absorption from the respiratory epithelial surface through aquaporins and the paracellular pathway. ASL dehydration contributes to mucus hyperviscosity and restricts cilia function as well as MCC. Mucus plugging predisposes the lungs to cycles of chronic infection and inflammation leading to lung function decline [29]. Osmotic agents, such as inhaled hypertonic saline (HS) or mannitol, allow water to be drawn into the airway surface and reverse dehydration by Na^+^ hyperabsorption via the ENaC channel. Both these treatments increase the forced expiratory volume in 1 s (FEV1) and improve the MCC in CF patients [30,31]. CFTR modulators, which mainly act as correctors and potentiators increasing functional CFTR at the epithelial cell apical surface, improve ion channel function and therefore airway hydration [15]. It seems that alternative strategies based on the activation of non-CFTR Cl^−^ channels, such as ANO1, constitute potential therapeutic approaches. Direct or indirect ENaC blockers have the potential to increase ASL hydration by inhibiting Na^+^ absorption via the ENaC channel, but their efficacy has yet to be demonstrated. However, the loss of the CFTR protein inhibitory effect on ENaC and the Na^+^ hyperabsorption are controversial [32,33]. Increased sodium absorption has not been confirmed in airway epithelia of CF pigs [33], ferrets [34], and humans [35]. 

For a long time, research in CF has focused on abnormal Cl^−^ and Na^+^ transport. Yet, the CFTR protein also regulates HCO_3_^−^ [36], which was found to be a crucial factor in the pathophysiology of CF [9].

## 3. The CFTR Protein: Not Just a Chloride Channel

### 3.1. The CFTR Protein and Its Relationship with HCO_3_^−^ Transport

In addition to Cl^−^ transport, the CFTR protein is also permeable to HCO_3_^−^, but with a lower electrochemical gradient than for Cl^−^ ions [32]. Depending on the activity of the anion exchanger 2 (AE2 or SLC4A2) and electrogenic Na^+^/HCO_3_^−^ cotransporter (NBCe or SLC4A4) [18] expressed at the epithelial cell basolateral membrane, the CFTR protein also secretes HCO_3_^−^ in the airways, pancreas, salivary gland, intestine, and reproductive organs, with HCO_3_^−^ reabsorbed in the sweat duct [17,32,37]. HCO_3_^−^ plays an important role in controlling the pH of the fluid layers on the surface of epithelial cells where the CFTR protein is expressed. As lung involvement remains the main cause of morbidity and mortality in CF due to progressive pulmonary injury, this review focuses on this function in the lung. 

Correlations between the levels of CFTR expression, HCO_3_^−^ secretion, and host defense [29,38] have been demonstrated in the lungs and support that HCO_3_^−^ is an essential component of the ASL to maintain a normal ASL pH of ~7.1 [18,23] and promote a competent innate immunity, such as antimicrobial activity, in order to protect the lungs against infections. The antimicrobial peptides, proteins, and lipids in ASL, including lactoferrin, defensins, cathelicidins (LL-37), and secretory leukocyte peptidase inhibitor, exert individual as well as synergistic effects to rapidly kill bacteria [33,39]. Moreover, HCO_3_^−^ is essential to inhibiting bacterial growth, airway colonization, and biofilm formation [40]. In CF, these mechanisms are impaired by the decrease in ASL pH (about 0.5 units lower than normal ASL pH) [23], thereby compromising these antimicrobial defenses and promoting typical CF bacteria, such as *Staphylococcus aureus* [29,39,41]. In addition, HCO_3_^−^ secretion by the CFTR protein buffers H^+^ secretion by ATP12A (H^+^/K^+^-ATPase) at the apical membrane and therefore increases ASL acidity in CF, which also results in impaired antimicrobial activity [42].

MCC is complex and its effectiveness depends on the mucus that traps inhaled particles or pathogens, on the cilia whose beating removes secretions from the lungs, and on the PCL that protects the epithelium against dehydration and allows the cilia to beat [18]. As the CFTR protein is also expressed in the mucin granules of airway epithelial cells that contribute to mucus viscosity [43], it regulates mucus rheology as well as the viscoelastic properties of ASL and thus the MCC, with its key function in innate immune defense. Indeed, ASL alkalization is required to maintain low mucus viscosity [44] and support HCO_3_^−^ contribution. ASL volume and composition along with the coordinated cilia beat from the distal to proximal airways allow MCC that is an essential component of innate immune defense, protecting the lungs from inhaled agents including respiratory pathogens [15]. In CF patients, the mucus is thick and sticky. The loss of functional CFTR protein results in a dehydrated airway surface and in the production of viscous, acidic, muco-purulent secretions that are difficult to clear. However, studies carried out in piglets [45,46] show that the impaired MCC in CF is not linked to the reduced depth of the PCL caused by ASL dehydration [47,48]. Indeed, mucus viscosity depends on the mucins that require HCO_3_^−^ [18]. During the process of mucin release from intracellular granules, calcium (Ca^2+^) and hydrogen (H^+^) ions must be removed to enable the negatively charged mucins to expand by up to 1000-fold and form extracellular mucus gel networks. HCO_3_^−^ in the extracellular environment is critical for sequestering Ca^2+^ and H^+^ from the mucin anions to form complexes with these cations (H_2_CO_3_, CaHCO_3_^+^, and CaCO_3_) and to allow normal expansion of mucins. These ionic changes lead to a high internal osmotic gradient, with subsequent influx of water [49,50]. Quinton et al., suggested that as defective HCO_3_^−^ secretion in CF impairs calcium removal and prevents normal mucin expansion in the ducts or on the luminal surfaces of affected secretory organs, the mucins tend to remain aggregated, poorly solubilized, and less transportable [50,51]. This results in increased mucus viscosity, adhesion, and stasis, leading to impaired MCC [19,45,52]. This mechanism is supported by animal studies showing that high concentrations of HCO_3_^−^ delivered to the airways of CF rats and pigs changed the proportion of condensed/expanded mucins and increased the MCC [53,54]. Moreover, pH values below 7.0 also decreased the ciliary beating in vitro [55] and contributed to the impaired MCC through increased mucus thickness. 

Finally, SPLUNC1, in addition to preventing ASL dehydration, is an antimicrobial protein [23]. In CF, it is degraded by neutrophil elastase, which is present in high concentrations in the sputum and is ineffective at ASL pH values below 7.0 [19,24], also leading to reduced activity of Gram-negative bacteria, such as *Haemophilus influenzae*, *Pseudomonas aeruginosa* and the *Burkholderia cepacia* family, in CF airways [23].

### 3.2. The CFTR Protein and Its Relationship with GSH and SCN^−^

Reactive oxygen species (ROS) can be generated by exogenous sources, such as pollution and cigarette smoke, or be produced endogenously by inflammatory cells, including macrophages and neutrophils, when the pathogen enters into the airways. While endogenous ROS are important for the antimicrobial host defense, their increase, as observed in CF due to inflammatory cells (in particular neutrophils) and bacteria in the airways, can lead to cell death and tissue damage defined as oxidative stress [12]. In addition to Cl^−^ and HCO_3_^−^, the CFTR protein has also been shown to be involved in the transport of GSH and SCN^−^ in the apical membrane and allows maintaining appropriate GSH and SCN^−^ levels in the ASL. Both molecules, when present in high concentrations in the ASL, play an important role as natural antioxidants, especially in the lungs [56,57]. GSH is a sulfhydryl-rich tripeptide that represents the frontline defense of the lungs against oxidative stress-induced damage. Through a reaction catalyzed by glutathione peroxidase, two GSH molecules form oxidized glutathione (GSSG) and free hydrogen peroxide (H_2_O_2_) is reduced to water. The GSH:GSSG ratio is considered as a biomarker of oxidative stress [58]. SCN^−^ is an anion which also plays an important protective role in the lungs. It can reduce potentially harmful levels of H_2_O_2_ and hypochlorite (OCl^−^). First, tissue-innocuous hypothiocyanite (OSCN^−^) produced from HCN^−^ by H_2_O_2_ lactoperoxidase catalysis consumes H_2_O_2_ and has been shown to have antimicrobial activity [56]. Second, OCl^−^ production by myeloperoxidase released from white blood cells (WBC) can be limited by SCN^−^ that competes with Cl^−^ for this enzyme. Third, SCN^−^ can also reduce OCl^−^ without catalysis [59].

An imbalance between antioxidants and ROS leads to oxidative stress. CF lung injury is characterized by an exaggerated neutrophil-dominated inflammation that is ineffective to clear bacterial infections and the oxidative stress. Indeed, the loss of functional CFTR protein reduces the GSH [60] and SCN^−^ [57] concentrations in the ASL. As a consequence, the oxidative stress is exacerbated and causes pulmonary damage. Moreover, OCl^−^ overproduction in the absence of adequate SCN^−^ leads to WBC self-destruction, which in turn results in pulmonary injuries by additional destructive agents [59]. Some clinical trials using GSH and SCN^−^ have been carried out in CF, but their results remain controversial.

### 3.3. The CFTR Protein and Its Relationship with Immune Cells

Beside its role in innate immunity related to ion transport, the CFTR protein is also expressed in immune cells, including macrophages and neutrophils [61]. Due to their plasticity, alveolar macrophages (AMs) help maintain immunological and physiological homeostasis in the lungs and are the front line of cellular defense against pathogens that were not eliminated by the mechanical defenses of the airways [62]. They phagocytize and kill infectious organisms within endocytic vacuoles that are rich in ROS, lysozymes, antimicrobial peptides, and proteolytic enzymes [63]. They also allow inflammatory response initiation and recruitment of activated neutrophils into the alveolar spaces through synthesis and secretion of cytokines and chemokines, such as interleukin (IL)-1, IL-6, IL-8, and tumor necrosis factor (TNF)-α, as well as AA metabolites (leukotrienes and prostaglandins). Airway inflammation in CF is therefore characterized by increased levels of pro-inflammatory cytokines and chemokines, as well as a massive influx of neutrophils that, through the release of ROS and proteases, cause respiratory epithelium damage and progressive lung injury. Despite the intense inflammatory response in CF, neutrophils and macrophages are paradoxically ineffective in clearing bacterial infections and resolving the inflammation. Indeed, impaired immune cellular defense has been reported in CF. As macrophages and neutrophils express functional CFTR, the dysregulation of their functions in CF could be a consequence of CFTR protein dysfunction. However, the lung environment in CF (hydration and rheology of mucus, ROS, proteases, etc.) can also affect the ability of macrophages to properly respond to inflammatory triggers or efficiently phagocytize pathogens [62].

Macrophages in CF show multiple defects, including an ineffective uptake of pathogens due to dysregulated phagocytic and/or signaling receptors and a decreased efferocytosis, phagocytosis, ROS production, and bacterial killing [64]. CFTR can actually also regulate the signaling of receptors involved in the recognition of microbial stimuli called pathogen-associated molecular patterns (PAMPs). Toll-like receptors (TLRs), a member of the pattern recognition receptors (PRR), partially mediate the inflammation by activating the nuclear factor (NF)-κB, which governs a molecular pathway that induces the production of inflammatory mediators [65]. Moreover, reduced CFTR expression in AMs of mice resulted in increased NF-κB activation and IL-8 secretion [66]. Efferocytosis is the process by which apoptotic cells are removed in acute or chronic inflammation and it is therefore crucial to inflammation resolution and lung homeostasis restoration [62,63]. It is predominantly mediated by macrophages. Disruption of this mechanism leads to secondary necrosis of accumulating apoptotic cells, release of necrotic cell debris called damage-associated molecular patterns (DAMPs), and uncontrolled activation of the innate immune system. In CF, in addition to the accumulation of apoptotic cells in the airways, the macrophages show delayed efferocytosis due to reduced expression of the phosphatidylserine receptor, which undergoes proteolytic cleavage by the neutrophil elastase [67] present in the CF airways. Thus, the inflammation is self-sustaining and causes tissue damage [63]. Phagocytosis allows removal of pathogens, dead cells, and debris, with AMs also being involved in surfactant recycling. It is a complex mechanism, which is mediated by the fusion of phagosomes generated by apical membrane invagination with lysosomes to form acidic phagolysosomes that facilitate bacterial killing [63]. It depends on a complex transcriptional response, which includes cytoskeleton dynamics and vesicle trafficking/fusion, lysosomal enzyme production, and proton-pump acidification of lysosomal compartments [62]. In CF, phagocytosis is greatly decreased, which could explain the defective elimination of pathogens, such as *Pseudomonas aeruginosa* and *Burkholderia cenocepacia*. The loss of functional CFTR protein in CF affects this bacterial killing by macrophages. Di et al., reported that phagolysosomes in AMs from CFTR^−/−^ mice were ∼2 pH units more alkaline. They suggested that the CFTR protein contributes to lysosomal acidification and that its dysfunction in CF prevents phagolysosome acidification, thereby providing an environment favorable to bacterial replication [68]. As CFTR dysfunction in macrophages affects their lipid metabolism and membrane structure, it has been proposed that an imbalance in the activity of pH-sensitive enzymes (acid sphingomyelinase and ceramidase) involved in ceramide metabolism may explain the link between dysfunctional CFTR protein and the defective bacterial killing capacity of macrophages. This aspect will be discussed in the review on the relationship between the CFTR protein and metabolism of lipids. However, this role of the CFTR protein in controlling phagosomal pH is controversially discussed. Other authors showed that CFTR-deficient macrophage cell lines and primary mouse and human AMs display no change in the pH of phagosomes, partly related to the methods used to quantify phagolysosomal pH [69].

### 3.4. The CFTR Protein and Its Relationship with Lipid Metabolism 

The CFTR protein is present within a complex lipid bilayer forming the plasma membrane. The interactions between the CFTR protein and this membrane and therefore the metabolism of lipids still need to be clarified. Disturbances in the metabolism of lipids have been described in CF, along with their role in lung CF pathophysiology. An abnormal essential fatty acid (EFA) profile has been documented in the blood and tissues of CF patients, including moderately decreased concentrations of linoleic acid (LA, 18:2 ω-6) and docosahexaenoic acid (DHA, C22:6 ω-3), associated with increased concentrations of AA (C20:4 ω-6) [70,71]. The increased omega-6/omega-3 ratio and AA concentrations lead to a proinflammatory state [72]. As this imbalance has also been reported in well-nourished CF patients [72], other mechanisms have been suggested by Peretti et al., including excessive oxidation of EFAs as an energy source, increased metabolism of LA to AA associated with increased expression and activation of Δ5- and Δ6-desaturase enzymes, increased production of eicosanoids linked to inflammatory responses, higher lipid turnover in cell membranes, defective lipid incorporation into the plasma membrane, as well as ceramide deficiency [73,74]. The more marked EFA deficiency in severe CF genotypes [75] reinforces the role of the basic CF defect in EFA metabolism disturbances via the 5′ AMP-activated protein kinase (AMPK) signaling pathway [76,77]. Indeed, the CFTR protein is also associated with kinases and phosphatases that activate other signaling pathways. The loss of functional CFTR protein leads to an impaired Ca^2+^ metabolism and thus to an increased intracellular Ca^2+^ that activates Ca^2+^/calmodulin-dependent protein kinase kinase β (CaMKKβ), which in turn activates AMPK. Increased AMPK activity indirectly stimulates the expression and activity of Δ5- and Δ6-desaturase, whereas CaMKKβ or AMPK inhibition reduces desaturase expression [77]. These abnormalities in EFA contribute to chronic and uncontrolled inflammation and therefore to irreversible lung damage through the production of eicosanoids derived from AA from neutrophils and AMs. Among these eicosanoids, leukotriene (LT)B-4 is a potent neutrophil chemoattractant that amplifies the neutrophilic inflammation in CF patients [71,77,78]. 

Cholesterol is an essential constituent of cell membranes, representing about 10–45% of the lipid bilayer in mammalian cells. It not only contributes to controlling the membrane fluidity and lipid compartmentalization, but also modulates the function of membrane proteins, including ion channels. Plasma levels of cholesterol appear to be low in CF patients. The loss of CFTR function increases the cholesterol content of the plasma membrane, which suggests an inherent defect in intracellular cholesterol transport in CF [79].

Sphingolipids are another major component of the plasma membrane and, together with cholesterol, they form microdomains called lipid rafts. As signaling molecules, these interconvertible bioactive lipids contribute to various cell function processes. Cell interaction, adhesion, proliferation, migration, and death are in part regulated by sphingolipids. In addition to being part of the plasma membrane, ceramide, sphingosine, and sphingosine-1-phosphate (S1P), which are the key molecules of sphingolipid signaling, are involved in cell processes and pathological processes, such as inflammation, as well [80]. As mentioned above, sphingolipids play an important role in the pathophysiology of CF. Cellular ceramide concentrations are controlled by the de novo synthesis of ceramide, production of ceramide by the salvage pathway, and activity of the pH-sensitive enzymes (acid sphingomyelinase and ceramidase) [81]. However, the role of ceramide in CF is still controversial. Some authors have shown that ceramide is increased in CF epithelial cells, while others have reported that its concentration is reduced. These discrepancies may be accounted for by the method of analysis, the use of different antibodies (monoclonal versus polyclonal), and the ceramide species detected by each of the antibodies [74,81]. This ceramide imbalance is not dependent on diet [81]. The ratio of long-chain to very long-chain ceramides (LCC/VLCC) seems to be of importance [63,81,82]. Indeed, ceramide is a sphingolipid implicated in pro-inflammatory and pro-apoptotic signaling, as well as in proliferative signaling. LCCs are pro-inflammatory, while VLCCs are anti-inflammatory. In CF, airway epithelial cells show an accumulation of LCCs compared to VLCCs [81,83]. Horati et al., demonstrated that this increased LCC/VLCC ratio in the broncho-alveolar lavage of CF infants was correlated with the PRAGMA-CF chest computed tomography score and with various pro-inflammatory markers and could therefore predict structural lung disease in CF children [83]. In addition to its role in cell processes as other sphingolipids, sphingosine, which is released from ceramide through ceramidase activity, is also involved in bacterial killing, bacterial membrane permeabilization, intracellular ATP release, decreased metabolic activity, and reduced bacterial survival. In CF, sphingosine concentrations are decreased in airway epithelial cells due to the repressed acid ceramidase activity, which contributes to the high infection susceptibility to CF pathogens, such as *Staphylococcus aureus* and *Pseudomonas aeruginosa*. This bactericidal activity of sphingosine itself is also pH dependent and is profoundly reduced at alkaline pH [80]. Sphingosine is phosphorylated into S1P by sphingosine kinases. S1P is involved in a number of cellular processes, including proliferation, apoptosis, lymphocyte egress, endothelial barrier function, angiogenesis, and inflammation. CFTR could be involved in the cellular uptake of S1P. In CF, the loss of functional CFTR is actually associated to a decreased S1P intake. Yet, a higher availability of S1P allows the interaction with the S1P receptor and its signaling towards proliferation, migration, angiogenesis, and inflammation, which are responsible for fibrosis and remodeling in CF [84]. 

On the other hand, lipids could affect the CFTR protein stability and function by various mechanisms, such as direct interaction, changes in membrane localization, membrane forces, or initiation of a signaling cascade [81] As the CFTR protein clusters into lipid rafts, homeostasis of lipids is important and their imbalance contributes to the pathophysiology of CF. This imbalance in the plasma membrane can affect the membrane microdomains, membrane trafficking, as well as activity of membrane-associated transport and receptor systems that control inflammation; as such, it can affect the activity of the CFTR protein, but the precise mechanism is still incompletely understood [81,82,84]. In order to develop new treatments for CF, it may therefore be useful to understand the mechanisms by which the loss of functional CFTR protein leads to this imbalance in the metabolism of lipids and how it affects the disease as well as the interactions between the CFTR protein and lipids in plasma membrane.

### 3.5. The CFTR Protein and Its Relationship with the Pathophysiology of CF

**Figure 3:**
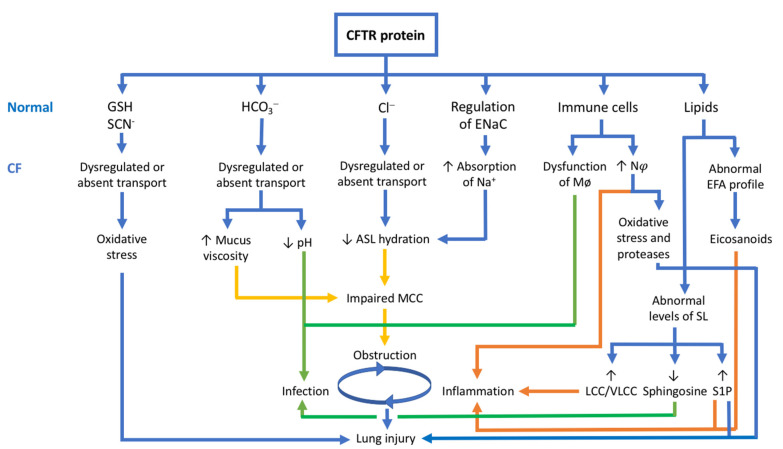
Relationship between CFTR protein and lung pathophysiology of CF. Modified from Elborn [1]. The CFTR protein is not just a chloride channel. It is also involved in HCO_3_^−^, GSH and SCN^−^ transport, regulation of ENaC, immune cells, and metabolism of lipids. In CF, the loss of functional CFTR results in bronchial obstruction (in yellow), inflammation (in orange), and infection (in green), leading to lung injury. Abbreviations: CFTR = cystic fibrosis transmembrane conductance regulator; Cl^−^ = chloride; HCO_3_^−^ = bicarbonate; GSH = glutathione; SCN^−^ = thiocyanate; Na^+^ = sodium; ENaC = epithelial sodium channel; ASL = airway surface liquid; Mø = macrophages; N*φ* = neutrophils; SL = sphingolipids; S1P = sphingosine-1-phosphate; LCC/VLCC = long-chain ceramides/very long-chain ceramides.

As the pulmonary disease remains the main cause of morbidity and mortality in CF, this review focuses on lung pathophysiology, which is characterized by bronchial obstruction, infection, and inflammation leading to bronchiectasis and progressive pulmonary injury [7,8]. Figure 3 summarizes the relationship between the CFTR protein functions presented in this review article and the lung pathophysiology of CF.

Airway obstruction in CF is due to mucus dehydration/hyperviscosity, with subsequent impaired MCC resulting from dysregulated Cl^−^, HCO_3_^−^ and Na^+^ transport caused by the loss of functional CFTR protein. This abnormally thick and sticky mucus promotes chronic infection and inflammation. While chronic bacterial infection has long been recognized as the main stimulus for airway inflammation in CF, increasing evidence suggests that the inflammatory response in CF lungs occurs early in the disease course, before any infection, and supports the crucial role of the CFTR protein [85,86]. As shown by studies in CF ferrets and CF infants and young children, mucus obstruction and neutrophilic inflammation are present even in the absence of detectable bacterial infection [87,88,89], which implies that mucus obstruction in CF can trigger sterile airway inflammation. Balázs et al., suggested that hypoxic epithelial cell necrosis due to the thick and sticky mucus can lead to IL-1α release in CF airways. Activation of this IL-1 signaling pathway results in the expression of chemokines, such as IL-8 that recruits neutrophils into the airways [89]. Afterwards, bacterial infection activates immune cells, such as neutrophils, macrophages, and dendritic cells, by both PAMPs and DAMPs. This contributes to the synthesis and secretion of cytokines and chemokines, such as IL-1, IL-6, IL-8, and TNF-α, as well as leukotrienes and prostaglandins from AA metabolism and maintains the inflammatory response. In CF, neutrophils represent the major immune cell type in this inflammatory process. While neutrophils play an essential role in antibacterial host defense, they also produce ROS and proteases, resulting in lung damage and a self-perpetuating cycle of inflammation. T cell activation as part of the adaptive immune response leads to the secretion of IL-17, a cytokine that regulates neutrophil recruitment, thereby perpetuating the inflammatory response by more Th17 cells [90]. Furthermore, the transcription factor NF-κB is overactivated in CF, leading to the production of inflammatory cytokines, such as IL-8, and the recruitment of neutrophils. Bacterial PAMPs enhance NF-κB activation by activating TLR signaling and myeloid differentiation factor 88 (MyD88) [91].

Despite this intense neutrophilic response, CF is characterized by an enhanced lung susceptibility to infections and a defective bacterial eradication due to dysfunctional CFTR protein. Indeed, in CF, at least two important airway defenses, i.e., the MCC and innate immunity system, are impaired, notably due to a dysregulated HCO_3_^−^ secretion. These findings support that preventive measures should be initiated early in CF [29,52]. However, proximal airway defects, characterized by smaller and hypo-distended lumens as compared to non-CF airways, have been described before the onset of lung disease in CF mice and pigs, suggesting an origin during branching morphogenesis [92]. The lack of CFTR-dependent anion transport/liquid secretion likely contributes to abnormal development of cartilaginous airways that may explain early airflow obstruction and air trapping in CF [92,93,94]. Therefore, lung disease in CF is present very early or even at birth [29].

## 4. Conclusions

CF is caused by mutations in a single gene encoding a multifunctional protein. As shown in this review article, the various functions of the CFTR protein are crucial for a number of physiological processes and due to its wide distribution, the loss of functional CFTR protein in CF leads to a large number of metabolic dysfunctions and explains the complexity of CF. Even in the era of CFTR modulators targeting the CFTR protein, it remains essential to improve our understanding of the CF pathophysiology and develop new effective and safe drugs.

## Figures and Tables

**Table 1:** Affected organs and manifestations in cystic fibrosis.

Affected Organs	Manifestations
Reproductive tract	Absence of vas deferens, male (female) infertility
Sweat glands	Elevated sweat chloride
Lungs	Airway obstruction, chronic bacterial infection, bronchiectasis, pneumothorax, hemoptysis
Sinuses	Sinusitis, polyps
Pancreas	Exocrine insufficiency, cystic fibrosis-related diabetes
Liver	Obstructive biliary tract disease
Intestine	Meconium ileus, distal intestinal obstruction syndrome, rectal prolapse

## Data Availability

Not applicable.
